# Negative feedback between TAp63 and Mir-133b mediates colorectal cancer suppression

**DOI:** 10.18632/oncotarget.13515

**Published:** 2016-11-23

**Authors:** Jing Dai, Hao Wu, Yi Zhang, Kai Gao, Gui Hu, Yihang guo, Changwei Lin, Xiaorong Li

**Affiliations:** ^1^ Department of Gastrointestinal and Thyroid Surgery, The Third Xiang Ya Hospital of Central South University, Changsha, Hunan 410013, P. R. China

**Keywords:** TAp63, miR-133b, negative feedback, proliferation, metastasis

## Abstract

**Background:**

TAp63 is known as the most potent transcription activator and tumor suppressor. microRNAs (miRNAs) are increasingly recognized as essential components of the p63 pathway, mediating downstream post-transcriptional gene repression. The aim of present study was to investigate a negative feedback loop between TAp63 and miR-133b.

**Results:**

Overexpression of TAp63 inhibited HCT-116 cell proliferation, apoptosis and invasion via miR-133b. Accordingly, miR-133b inhibited TAp63 expression through RhoA and its downstream pathways. Moreover, we demonstrated that TAp63/miR-133b could inhibit colorectal cancer proliferation and metastasis in vivo and vitro.

**Materials and Methods:**

We evaluated the correlation between TAp63 and miR-133b in HCT-116 cells and investigated the roles of the TAp63/miR-133b feedback loop in cell proliferation, apoptosis and metastasis via MTT, flow cytometry, Transwell, and nude mouse xenograft experiments. The expression of TAp63, miR-133b, RhoA, α-tubulin and Akt was assessed via qRT-PCR, western blot and immunofluorescence analyses. miR-133b target genes were identified through luciferase reporter assays.

**Conclusions:**

miR-133b plays an important role in the anti-tumor effects of TAp63 in colorectal cancer. miR-133b may represent a tiemolecule between TAp63 and RhoA, forming a TAp63/miR-133b/RhoA negative feedback loop, which could significantly inhibit proliferation, apoptosis and metastasis.

## INTRODUCTION

The transcription factor p63 is a member of the p53 gene family that plays a complex role in cancer due to its involvement in tumor suppression [1]. Through two distinct promoters, P1 and P2, the p63 gene generates the transactivating TAp63 isoform and the inhibitory DNp63 isoform [2]. TAp63 is related to cell-cycle arrest and apoptosis [3]. By regulating the expression of TAp63, many genes could take part in tumor development [4]. Furthermore, TAp63 is known as the most potent transcription activator and tumor suppressor [5]. It can activate a large number of downstream targets that collectively repress tumor formation [6, 7]. In addition to the numerous protein-coding targets of TAp63, microRNAs (miRNAs) are increasingly recognized as essential components of the p63 pathway, mediating downstream post-transcriptional gene repression [8–10].

miRNAs are a type of 18- to 24-nucleotide regulatory noncoding RNA molecules [11]. miRNAs regulate gene expression via post-transcriptional gene silencing of messenger RNAs (mRNAs), potentially leading to mRNA degradation, with consequent inhibition of gene translation [12]. In gastric cancer, miR-133b acts as a tumor suppressor and negatively regulates FSCN1 expression [13]. Restoring the expression of miR-133b can inhibit the growth and invasion of colorectal cancer cells via directly targeting EGFR [14]. miR-133b can also significantly inhibit bladder cancer cell proliferation and apoptosis by targeting Bcl-w [15]. All of these findings imply functional significance of miR-133b deficiency in tumorigenesis and suggest that miR-133b plays an important role in the tumor suppressor network.

Transcriptional regulation has been indicated as one of the most important steps in the synthesis of miRNAs [16–18]. A previous study by our group revealed that miR-133b functions as transcriptional target of TAp63, and downregulation of TAp63 is one of the main causes of low expression of miR-133b in colorectal cancer [19]. In addition, we reported that there is a great deal of crosstalk between p63 and the microRNA network based on a literature analysis and predicted approximately 39 pairs of p63-miRNA feedback, including TAp63/miR-133b [20]. The aim of this study was to investigate a negative feedback loop between TAp63 and miR-133b that is at least partly due to miR-133b-mediated RhoA repression.

## RESULTS

### Overexpression of TAp63 inhibited CRC cell proliferation, apoptosis and microtubule formation

**Figure 1:**
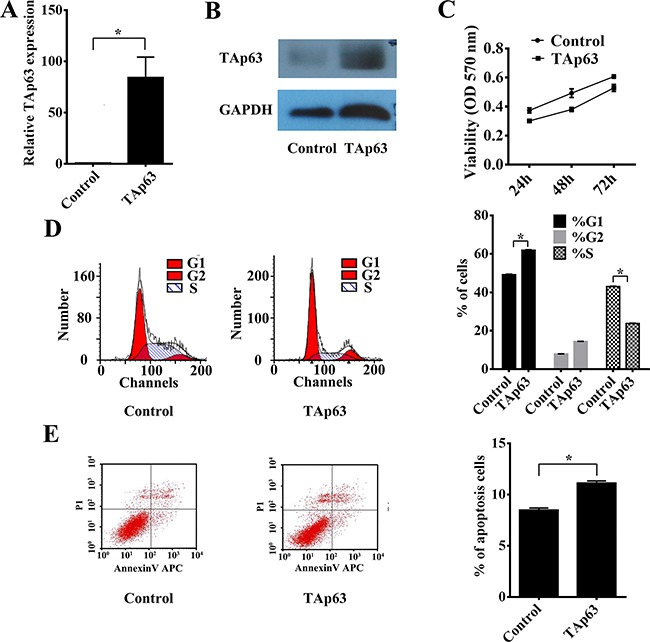
Overexpression of TAp63 inhibits cell proliferation, apoptosis and microtubule formation HCT-116 cells were transfected with the TAp63-expressing vector or negative controls (NCs). The expression of TAp63 was analyzed via qRT–PCR **A**. and western blotting **B**. **C**. The growth curves of the cells transfected with the TAp63-expressing vector or NCs were compared using the MTT assay. *P < 0.05. **D**. Distribution of cells in three phases (G1, S, and G2) of the cell cycle, as determined by flow cytometry analysis. Cytometric quantification of the experiments described in the chart, showing the proportions of cells in G1, S, and G2 phases.*P < 0.05. **E**. The apoptosis assay shows that the apoptosis rate in HCT-116/TAp63 cells was significantly higher than in the control. *P < 0.05.

To determine the impact of TAp63 on the growth of CRC cells, we constructed a TAp63 plasmid and used qRT–PCR and western blotting to confirm the expression of TAp63. We obtained pooled HCT-116 clones (HCT-116/TAp63 cells) that stably expressed TAp63 through G418 screening. The level of TAp63 was increased approximately 84-fold in HCT- HCT-116/TAp63 cells compared with the control vector group (Figure 1A and 1B). Then, the HCT-116/TAp63 cells were employed to explore the effects of TAp63 on cell growth using the MTT assay. As shown in Figure 1C, significant growth arrest was observed in HCT-116/TAp63 cells. Cell cycle analysis demonstrated that the expression of TAp63 induced a significant increase in the number of HCT-116/TAp63 cells in G1 phase, which was accompanied by a significant decrease in the number of HCT-116/TAp63 cells in S phase compared with the control cells (Figure 1D, *P<0.05). Moreover, overexpression TAp63 also increased apoptosis, as measured through FACS analysis of cells with annexin V and propidium iodide staining (Figure 1E, *P<0.05).

**Figure 2:**
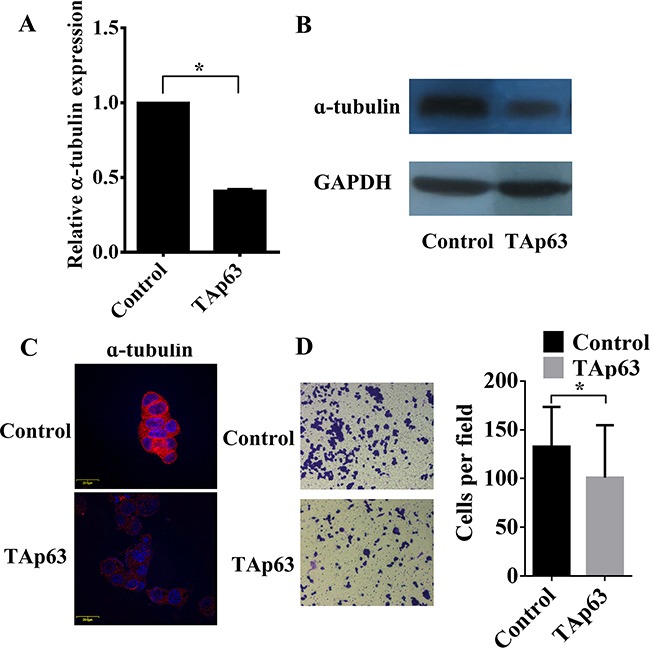
TAp63 regulates microtubule formation and invasion HCT-116 cells were transfected with the TAp63-expressing vector or negative controls (NCs). The expression of α-tubulin was analyzed via **A**. qRT–PCR, **B**. western blotting and **C**. immunofluorescence. **D**. Transwell invasion assays of HCT-116 and SW-620 cells transfected with either TAp63 or NC. After 48 h, invasive cells were counted in five random high-power fields. All of the data represent the means±sd of three different experiments analyzed in triplicate. *P<0.05.

We have previously demonstrated that overexpression of TAp63 suppresses the expression of epithelial and mesenchymal markers [19]. The role of TAp63 in regulating epithelial and mesenchymal markers prompted us to examine its effects on microtubule proteins. Subsequent experiments indicated that overexpression of TAp63 decreased the level of α-tubulin in HCT-116/TAp63 cells (Figure 2A–2C) and significantly inhibited cell invasion (Figure 2D). These observations suggest that TAp63 may play an important role in EMT reversion.

### miR-133b is involved in the antitumor effects of TAp63

**Figure 3:**
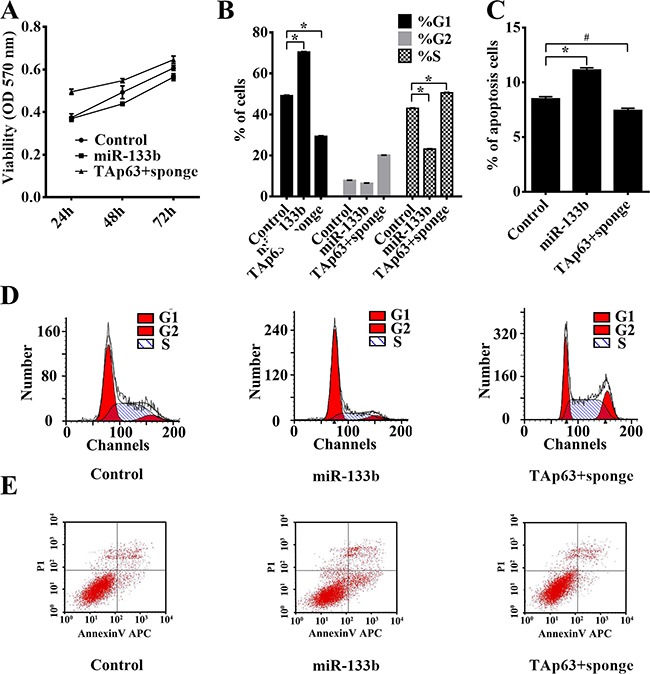
TAp63 inhibits cell proliferation through miR-133b HCT-116 cells were transfected with the miR-133b-expressing vector or the negative controls (NCs), or co-transfected with the TAp63-expressing vector and miR-133b sponge. **A**. The growth curves of the cells transfected with the miR-133b-expressing vector or NCs, or co-transfected with the TAp63-expressing vector and miR-133b sponge were compared using the MTT assay. *P < 0.05. **B**. Cytometric quantification of the cell cycle analysis experiments described in the chart, showing the proportions of cells in G1, S, and G2 phases.*P < 0.05. **C**. Apoptosis rates determined via apoptosis assays in HCT-116 cells transfected with the miR-133b-expressing vector or NC, or co-transfected with the TAp63-expressing vector and miR-133b sponge TAp63+sponge. *P < 0.05, ^#^*P*>0.05. **D**. Distribution of cells in three phases (G1, S, and G2) of the cell cycle, as determined by flow cytometry analysis. **E**. Apoptosis was measured via FACS.

A previous study by our group identified miR-133b as a transcriptional target of TAp63 [19]. We obtained pooled HCT-116 clones (HCT-116/miR-133b cells and HCT-116/TAp63+sponge cells) that stably expressed different levels TAp63 and miR-133b through G418 screening. Similar to the findings for TAp63, the MTS assay indicated that significant growth arrest occurred after transfection with vectors expressing miR-133b (Figure 3A). HCT-116/TAp63+sponge cells showed significantly increased proliferation compared with control group or HCT-116/miR-133b cells (Figure 3A). Cell cycle analysis demonstrated that overexpression of miR-133b induced a significant increase in the number of HCT-116/miR-133b cells in G1 phase, which was accompanied by a significant decrease in the number of HCT-116/miR-133b cells in S phase compared with the control cells (Figure 3B and 3D, *P<0.05), whereas co-transfection of TAp63 with miR-133b sponge accelerated the G1/S and S/G2 phase transitions (Figure 3B and 3D, *P<0.05). Moreover, overexpression miR-133b increased apoptosis, and miR-133b sponge reversed the effect of TAp63 on HCT-116 cell apoptosis (Figure 3C and 3E, *P<0.05).

**Figure 4:**
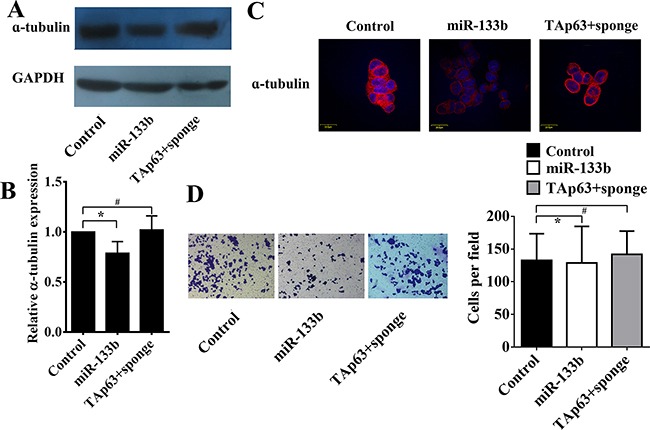
TAp63 regulates microtubule formation and invasion through miR-133b HCT-116 cells were transfected with the miR-133b-expressing vector or negative controls (NCs), or co-transfected with the TAp63-expressing vector and miR-133b sponge (TAp63+sponge). The expression of α-tubulin was analyzed via **A**. qRT–PCR, **B**. western blotting and **C**. immunofluorescence, *P < 0.05, ^#^*P*>0.05. **D**. Transwell invasion assays of HCT-116 and SW-620 cells transfected with either TAp63 or NC. After 48 h, invasive cells were counted in five random high-power fields. All of the data represent the means±sd of three different experiments analyzed in triplicate. *P < 0.05, ^#^*P*>0.05.

Similar to TAp63, overexpression of miR-133b also decreased the level of α-tubulin in HCT-116/miR-133b cells (Figure 4A–4C) and significantly inhibited cell invasion (Figure 4D, *P<0.05). Inhibiting the expression of miR-133b blocked the effect of TAp63 on α-tubulin and invasion (Figure 4A–4D).

**Figure 5:**
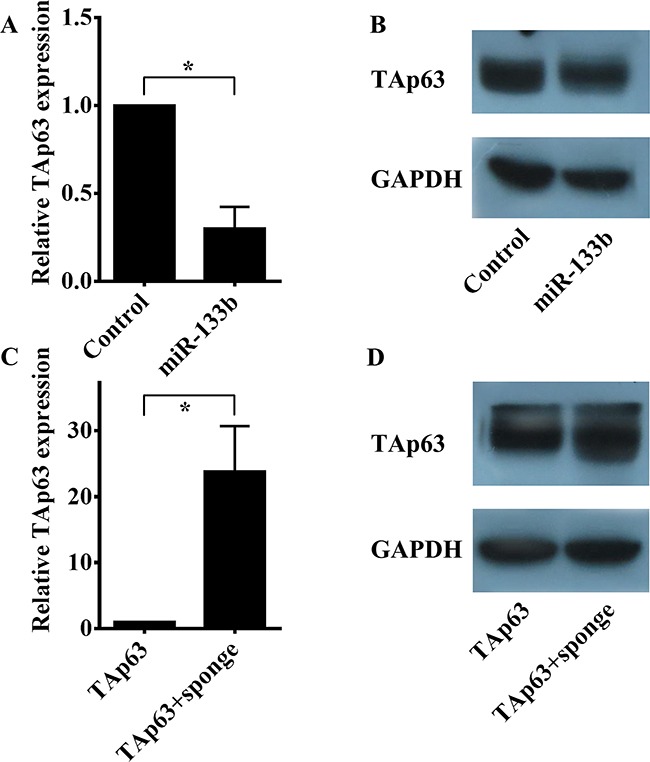
miR-133b regulates TAp63 expression **A**. qRT-PCR was performed to detect the expression levels of TAp63 following transfection with the miR-133b-expressing vector or negative controls in HCT-116 cells. The data are expressed as the means±sd. *P<0.05. **B**. Western blotting was performed to detect the protein expression levels of TAp63 following transfection with the miR-133b-expressing vector or negative controls in HCT-116 cells. **C**. qRT-PCR was performed to detect the expression levels of TAp63 following transfection with the TAp63-expressing vector or co-transfection with the TAp63-expressing vector and miR-133b sponge (TAp63+sponge) in HCT-116 cells. The data are expressed as the means±sd. *P<0.05. **D**. Western blotting was performed to detect the protein expression levels of TAp63 following transfection with the TAp63-expressing vector or co-transfection with the TAp63-expressing vector and miR-133b sponge (TAp63+sponge) in HCT-116 cells.

Interestingly, we found that the expression of TAp63 was decreased in HCT-116/miR-133b cells compared with the control group (Figures 5A and 5B, *P<0.05). Conversely, the expression of TAp63 was increased in HCT-116/TAp63+sponge cells compared with the HCT-116/TAp63 group (Figures 5C and 5D, *P<0.05). Therefore, we hypothesized that miR-133b can modulate its own transcriptional activator, TAp63, through a negative feedback mechanism.

### miR-133b modulates TAp63 via regulation of RhoA in a negative feedback mechanism

First, we hypothesized that miR-133b inhibits TAp63 expression by directly repressing its translation. Through computer analysis, we identified a potential binding site for miR-133b within the 3’ UTR of TAp63, extending from 165–186 bp (Supplementary Figure S1A). To test the hypothesis that miR-133b represses TAp63 through this site, we constructed a reporter vector consisting of luciferase cDNA followed by the 3’ UTR of TAp63. The results showed that TAp63 is not a potential miR-133b target in HCT-116 cells (Supplementary Figure S1B, ^#^*P*>0.05).

**Figure 6:**
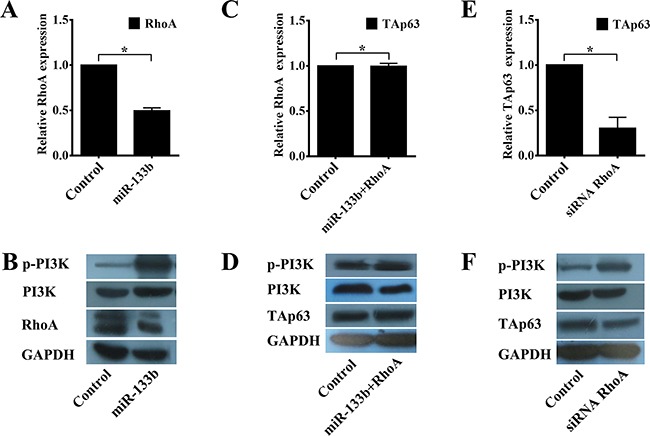
miR-133b regulates TAp63 expression through the RhoA and PI3K/Akt pathways **A**. qRT-PCR analysis of RhoA in HCT-116 cells transfected with the miR-133b-expressing vector or negative controls. The data are expressed as the means±sd. *P<0.05. **B**. Western blot analysis of RhoA and p-PI3K in HCT-116 cells transfected with the miR-133b-expressing vector or negative controls. **C**. qRT-PCR analysis of TAp63 in HCT-116 cells transfected with the negative controls or co-transfected with the miR-133b- and RhoA-expressing vectors. The data are expressed as the means±sd. ^#^*P*>0.05. **D**. Western blot analysis of TAp63 and p-PI3K in HCT-116 cells transfected with the negative controls or co-transfected with the miR-133b- and RhoA-expressing vectors. **E**. qRT-PCR analysis of TAp63 in HCT-116 cells transfected with RhoA siRNA or the negative controls. The data are expressed as the means±sd. *P<0.05. **F**. Western blot analysis of TAp63 and p-PI3K in HCT-116 cells transfected with RhoA siRNA or the negative controls.

Lin and Qin reported RhoA as a direct target of miR-133b in cervical and colorectal cancer [19, 21]. Inhibition of miR-133b significantly blocks the PI3K/Akt signaling pathway by targeting RhoA [22], and inhibiting the activation of PI3K/Akt signaling induces the expression of TAp63 [23]. Thus, we next examined the effect of RhoA on the expression of TAp63. As shown previously, HCT-116/miR-133b cells exhibited significantly lower expression of RhoA than the control cells (Figures 6A and 6B), and overexpression of miR-133b significantly increased the phosphorylation of PI3K (Figures 6A and 6B, *P<0.05). Co-transfection of the miR-133b-expressing vector with the RhoA-expressing vectors rescued the expression of TAp63 and PI3K (Figures 6C and 6D, ^#^*P*>0.05). Similarly, the phosphorylation of PI3K in HCT-116 cells transfected with the RhoA siRNA vector was significantly higher than in the control (Figures 6E and 6F, *P<0.05), whereas the expression of TAp63 was significantly decreased (Figures 6E and 6F, *P<0.05). The effectiveness of RhoA knock-down were shown in Supplementary Figure S2. Although miR-133b might have additional targets, the results of these experiments taken together suggest that miR-133b indirectly regulates TAp63 through RhoA.

### TAp63/miR-133b inhibit metastasis in vitro

**Figure 7:**
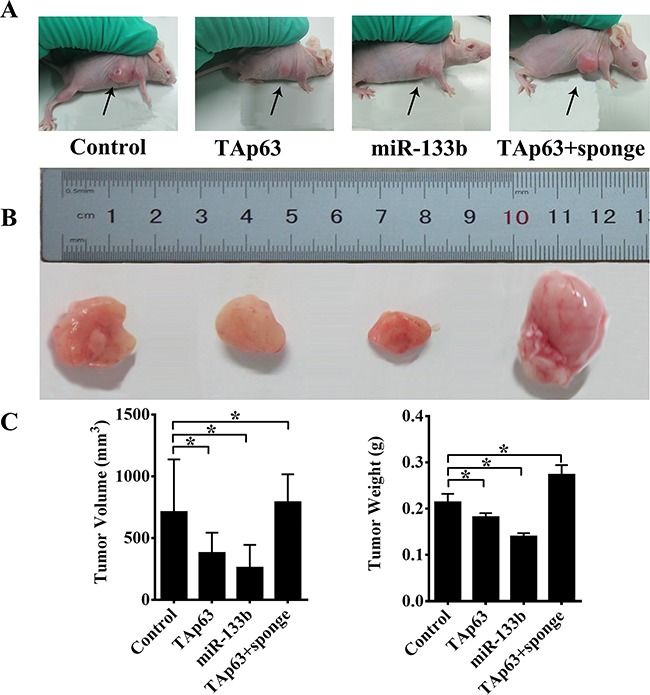
TAp63/miR-133b inhibits tumorigenicity in vivo **A**. Tumors formed in nude mice. HCT-116 cells stably transfected with the TAp63-expressing vector, miR-133b-expressing vector or negative controls (NCs) or co-transfected with the TAp63-expressing vector with miR-133b sponge (TAp63+sponge) were injected into the flanks of nude mice (n=5), and the mice were sacrificed after 4 weeks. **B**. Images of whole tumors from nude mice injected with HCT-116 cells stably transfected with the TAp63-expressing vector, miR-133b-expressing vector or negative controls (NC), or co-transfected with the TAp63-expressing vector and miR-133b sponge (TAp63+sponge). **C**. The average volume and weight of the tumors from the mice injected with HCT-116 cells stably transfected with the TAp63-expressing vector or the miR-133b-expressing vector were significantly lower than that in those injected with negative control cells. The data are expressed as the means±sd. *P<0.05.

We previously demonstrated that TAp63 inhibited colorectal cancer metastasis in vivo [19]. Thus, we next explored the role of TAp63/miR-133b in CRC metastasis in vitro. We obtained pooled HCT-116 clones (HCT-116/TAp63 cells, HCT-116/miR-133b cells and HCT-116/TAp63+sponge cells) that stably expressed different levels of TAp63 or miR-133b through G418 screening. Next, these cells and control cells were subcutaneously injected into nude mice. After 4 weeks, the subcutaneous tumors were harvested. In the various groups of five nude mice each, local cancers developed in all of the mice (Figure 7A). The average volumes of the subcutaneous tumors were 711.25±425.67 mm^3^ (HCT-116 cells), 379.85±163.74 mm^3^ (HCT-116/TAp63 cells), 260.32±184.96 mm^3^ (HCT-116/miR-133b cells) and 791.34±225.35 mm^3^ (HCT-116/TAp63+sponge cells) (Supplementary Table S1). The average weights of the subcutaneous tumors were 0.21±0.02 g (HCT-116 cells), 0.18±0.01 g (HCT-116/TAp63 cells), 0.14±0.01 g (HCT-116/miR-133b cells) and 0.27±0.02 g (HCT-116/TAp63+sponge cells) (Supplementary Table S1). The HCT-116/TAp63 group and HCT-116/miR-133b group showed significantly smaller tumors than the group treated with the scrambled sequence, indicating that TAp63 and miR-133b suppressed tumor growth (Figure 7B and 7C, *P<0.05). In contrast, the HCT-116/TAp63+sponge group showed significantly larger tumors than the group treated with the scrambled sequence (Figure 7B and 7C, *P<0.05).

**Figure 8:**
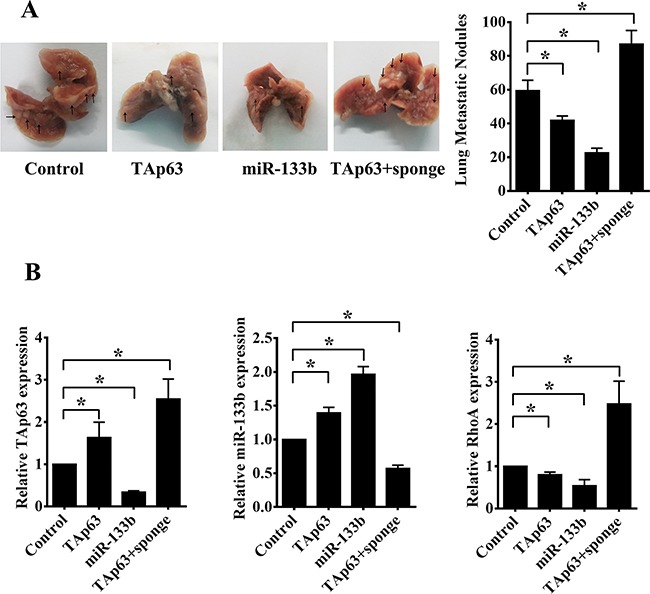
TAp63/miR-133b inhibits lung and liver metastasis **A**. Representative gross lung images from nude mice injected intravenously with HCT-116 cells stably transfected with the TAp63-expressing vector, miR-133b-expressing vector or negative controls (NC), or co-transfected with the TAp63-expressing vector and miR-133b sponge (TAp63+sponge) through the tail vein are shown, with metastases visible at the lung surface marked with bold black arrows. **B**. Liver tissue levels of TAp63, miR-133b and RhoA were measured via qRT-PCR in nude mice injected intravenously with HCT-116 cells stably transfected with the TAp63-expressing vector, miR-133b-expressing vector or negative controls (NC), or co-transfected with the TAp63-expressing vector and miR-133b sponge (TAp63+sponge) through the tail vein. *P<0.05.

To generate lung metastases, HCT-116 cells, HCT-116/TAp63 cells, HCT-116/miR-133b cells and HCT-116/TAp63+sponge cells were injected intravenously into four groups of mice through the tail vein. Metastasis nodules on the lung surface were detected in all mice. The average numbers of lung metastatic nodules were 59.40±6.23 (HCT-116 cells), 41.80±2.59 (HCT-116/TAp63 cells), 22.60±2.70 (HCT-116/miR-133b cells) and 87.00±8.12 (HCT-116/TAp63+sponge cells). The HCT-116/TAp63+sponge group showed significantly more lung metastatic nodules than the group treated with the scrambled sequence (Figure 8A, *P<0.05), whereas the average number of metastatic nodules in the lungs was dramatically decreased in the groups transfected with the TAp63- or miR-133b-expressing vector compared with the control groups (Figure 8A, *P<0.05).

We did not find any metastatic nodules on the liver surface. Therefore, we sought to determine whether any micrometastases occurred in the livers of the nude mice. The expression of human TAp63, miR-133b and RhoA mRNA in the livers of the mice was confirmed using human TAp63-, miR-133b- and RhoA-specific primers. Four groups of five nude mice each were analyzed, and all were observed to express human TAp63, miR-133b and RhoA in the liver. Consistent with previous results, the HCT-116/TAp63 group exhibited high levels of TAp63 and miR-133b and low levels of RhoA compared with the control group (Figure 8B). Additionally, the HCT-116/miR-133b group exhibited high levels of miR-133b and low levels of TAp63 and RhoA compared with the control group (Figure 8B), and the HCT-116/TAp63+sponge group exhibited high levels of RhoA and low levels of miR-133b compared with the control group (Figure 8B). Moreover, the expression levels of human TAp63 in the HCT-116/TAp63+sponge group were higher than in the HCT-116/TAp63 group (Figure 8B).

Taken together, these data demonstrate that negative feedback between TAp63 and miR-133b mediates colorectal cancer suppression.

## DISCUSSION

In a previous review, we described the p63/miRNA autoregulatory feedback loop and predicted approximately 39 pairs of p63-miRNA feedback in total [20]. Four of these p63-miRNA feedback-related pairs (ΔNp63/miR-130b, ΔNp63/miR-92, ΔNp63/miR-181a-5p and ΔNp63/miR-374a-5p) have been validated [24–27]. The major finding of this study is that TAp63/miR-133b mediates colorectal cancer suppression through inhibiting RhoA expression and pathways downstream of RhoA. We have previously shown that TAp63 regulates miR-133b [19], and the current study extends these results by suggesting the existence of a negative feedback loop in which TAp63 induces miR-133b, which inhibits TAp63 expression through RhoA and its downstream pathways. These results suggest that both TAp63 and miR-133b can function as tumor suppressor genes. These data also provide one mechanism by which the TAp63/miR-133b negative feedback loop can regulate cell proliferation and metastasis.

As the microRNA feedback regulation of p63 in cancer progression was systematically described in our previous study, we continued to study the crosstalk between TAp63 and miR-133b [20]. We were initially surprised to find that overexpression miR-133b could significantly downregulate TAp63 expression. Then, we analyze miR-133b binding to the 3’UTR of p63 via multiple components, including miRtarBase, microRNA.ORG and RNA22-HSA. Bioinformatic analyses revealed a potential target site. However, dual luciferase reporter gene assays demonstrated that miR-133b cannot target TAp63 mRNA directly. These results prompted us to look for a further mechanism through which miR-133b inhibits TAp63 expression. RhoA is a founding member of the Rho GTPase family, in which it is most readily recognized for its contributions to cell migration, the organization of the cytoskeleton, cell adhesion, progression through the cell cycle and gene expression [28–31]. It has been verified that miR-133b can bind a site within the 3′ UTR of RhoA to silence the mRNA [21], which we confirmed in a previous study [19]. The present study also showed that overexpression of miR-133b decreased RhoA levels. Furthermore, Lu et al. demonstrated that knockdown of RhoA expression enhanced the phosphorylation of Akt, similar to that induced by miR-133b [22]. Park et al. found that inhibiting the activation of PI3K/Akt signaling induced the expression of TAp63 [23]. Our results in the present study confirmed that RhoA inhibition, phosphorylation of PI3K and TAp63 expression were enhanced significantly when HCT-116 cells were transfected with an miR-133b-expressing vector. miR-133b act as a tiemolecule between TAp63 and RhoA. TAp63, miR-133b and RhoA form a negative feedback loop structure (TAp63/miR-133b/RhoA), in which TAp63 induces miR-133b, leading to repression of RhoA, and the PI3K/Akt pathway is finally activated, which will in turn suppress TAp63 expression, promote TAp63-related proliferation and inhibit TAp63-related apoptosis, thereby avoiding excessive apoptosis caused by TAp63. In contrast, when the expression of TAp63 is decreased in cancer cells, miR-133b expression is downregulated, and RhoA expression is increased, ultimately inhibiting the PI3K/Akt pathway, thus promoting TAp63 expression and TAp63-related apoptosis and inhibiting TAp63-related proliferation. Hence, this negative feedback loop acts as a protective mechanism in the cell physiology. Unfortunately, this protective mechanism is damaged in colorectal cancer by factors such as abnormal DNA methylation [32] or gene loss [33].

Interestingly, we found that stable overexpression of TAp63 and miR-133b could inhibit cell proliferation and apoptosis, whereas our previous study showed that TAp63 did not affect cell proliferation and apoptosis [19]. Jadhav V et al. reported a similar result and designed an experiment to explain this phenomenon [34]. Their results demonstrated that transient positive effects on growth could also be observed during early exponential culture. During later culture phases, however, the effects became more divergent. A possible explanation for these divergent results is that mRNA levels in cells during batch culture are subject to continuous changes, as is the culture environment [35]. In our previous experiments, cells were counted on consecutive days for 1 week using a hematocytometer [36], and this culture time is too long to maintain stable gene overexpression.

In conclusion, based on the results of the present study, we can deduce that the anti-tumor mechanism of TAp63 in colorectal cancer mainly involves the miR-133b-related pathway. In this pathway, miR-133b is an element in a feedback loop including TAp63 and RhoA, which greatly enhances the TAp63-related apoptosis signal and avoids abnormal proliferation in tumor cells.

## MATERIALS AND METHODS

### Ethics statement

This animal study was performed in strict accordance with the recommendations of the guidelines for the Care and Use of Laboratory Animals of the Third Xiang Ya Hospital of Central South University, China. The protocol was approved by the Institutional Animal Care and Use Committee of the Third Xiang Ya Hospital of Central South University. All efforts were made to minimize the suffering of animals.

### Cell culture and transfection

The human colon cancer cell lines HCT-116 was purchased from the Cell Center of the Xiangya School of Medicine, Central South University (Hunan, China). HCT-116 cells were cultured in H-Dulbecco's modified Eagle's medium (Gibco Life Technologies, USA) containing 10% fetal bovine serum (Gibco Life Technologies, USA) at 37˚C in humidified air with 5% CO_2_.

### Animals

BALB/C nude mice (N=40), aged 4–6 weeks, were purchased from the Hunan SJA Laboratory Animal Co., Ltd for use in all experiments. During the study, the animals were observed for any clinically relevant abnormalities daily. For the tumor proliferation experiment, 20 mice were randomly allocated into the following four groups, containing 5 mice each: negative control, TAp63, miR-133b and TAp63+sponge. Tumors were first generated in BALB/C mice by subcutaneously implanting 1×10^6^ HCT-116 cells in the right axillae of the mice. When the tumor volume reached 50 mm^3^, the tumors were measured twice a week, and the volume was calculated using the following formula: volume = width^2^× length × 0.52 [37]. All mice were monitored daily and euthanized after 4 weeks, followed by harvesting of the primary tumors. In the lung metastasis experiment, 20 mice were randomly allocated into the following four groups of 5 mice each: negative control, TAp63, miR-133b and TAp63+sponge. A total of 1×10^6^ HCT-116 cells suspended in 100 μL of PBS were injected intravenously into the mice through the tail vein [38]. All mice were monitored daily and euthanized after 4 weeks, and the lungs and liver were subsequently harvested. Visually detectable macroscopic lung metastases on the surface were counted [39].

### Quantitative real time-PCR analyses

Quantitative real time-PCR (qRT–PCR) was performed as described previously [19]. Briefly, qRT–PCR was conducted using the Real-Time Quantitative PCR SYBR Green detection reagent (Cowin Biotech Co., Ltd., China). miRNA qRT–PCR was performed using an All-in-One miRNA qRT-PCR Detection Kit (GeneCopoeia, USA). The relative expression of TAp63, RhoA and α-tubulin was normalized using the 2^-ΔΔCT^ method relative to GAPDH. The relative expression of miR-133b was normalized using the 2^-ΔΔCT^ method relative to U6-snRNA. All amplification reactions were run in triplicate. The primers sequences employed for PCR were as follows: p-Akt, sense5’-GCAGCACGTGTACGAGAAGA-3, antisense 5’-GGTGTCAGTCTCCGACGTG-3’ (67 bp) [40]. All the primers were synthesized by Yrbio Co.Ltd (Changsha, China).

### Western blot analysis and antibodies

Western blot analysis was performed as previously described using primary antibodies against the following proteins: p-Akt (Cell Signaling Technology, USA), total Akt (Cell Signaling Technology, USA), and α-tubulin (Abcam, UK). Briefly, cells were lysed in lysis buffer, followed by centrifugation at 14,000 g at 4°C for 10 min. The supernatants were then collected, and a BCA protein assay was performed. A volume of the extract equivalent to 100 mg of total protein was separated on a 10% polyacrylamide gel, followed by transfer to a PVDF membrane. The membranes were blocked for 2 h and then incubated with the primary antibodies at 4°C overnight. After washing with PBST, the membranes were incubated with an HRP-conjugated goat anti-rabbit IgG secondary antibody for 60 min at room temperature. The membranes were finally visualized with enhanced chemiluminescence reagents following exposure to X-ray film. All experiments were performed in triplicate.

### Immunofluorescence

Immunofluorescence detection was performed as described previously. Briefly, cultured HCT-116 cells were washed three times with PBS and blocked with 10% rabbit serum for 30 min. After washing with PBS, the HCT-116 cells were incubated with the primary antibodies overnight at 4°C. Then, the cells were incubated with Alexa Fluor 488 goat anti-rabbit IgG 30 for min and washed with PBS. Observations and photography were performed with an Olympus multifunction microscope (Olympus BX51, China).

### Luciferase assays

Luciferase assays were performed as described previously. The vectors were constructed by Yrbio Co. Ltd (Changsha, China). Briefly, HCT-116 cells were transiently transfected with the wild-type (Wt-TAp63) reporter plasmid containing potential miR-133b binding sites in the presence or absence of miR-133b using Lipofectamine 2000. Luciferase assays were performed 36 h post-transfection using a dual-luciferase assay system, and the results were normalized to the transfection efficiency with co-transfected Renilla luciferase. All experiments were performed in triplicate.

### MTT assays

Cell proliferation was determined using the Cell Proliferation Kit I (3-(4,5-dimethylthiazol-2-yl)-2,5-diphenyl tetrazolium bromide (MTT) (Sigma, USA) as previously described [14]. Briefly, approximately 1×10^4^ cells/well were grown in 96-well plates to 70–80% confluence. Then, MTT (10 mg/ml) was added to the cells, followed by incubation for 4 h, and the cells were then grown under the indicated conditions for 24 h, 48 h or 72 h [41]. A micro ELISA reader was used for quantification of the cells that survived oxidative stress at a wavelength of 492 nm.

### Cell cycle analysis

The cell cycle distribution was analyzed using a fluorescence-activated cell sorting (FACS) flow cytometer (FACSCalibur, Becton Dickinson). Briefly, we obtained pooled HCT-116 clones (HCT-116/TAp63 cells, HCT-116/miR-133b cells and HCT-116/TAp63+sponge cells) that stably expressed different levels of TAp63 and miR-133b through G418 screening. The cells were digested and subsequently fixed in 70% ethanol at 4˚C overnight. Then, the fixed cells (1×10^6^) were stained with propidium iodide, after which the cell cycle profiles could be assayed.

### Apoptosis analysis

We obtained pooled HCT-116 clones (HCT-116/TAp63 cells, HCT-116/miR-133b cells and HCT-116/TAp63+sponge cells) that stably expressed different levels of TAp63 and miR-133b through G418 screening. After an additional incubation for 72 h, the cells were harvested, stained with propidium iodide and an anti-annexin-V antibody, and analyzed using a FACS flow cytometer

### Transwell invasion assays

Transwell invasion assays were performed using a Transwell system (24 wells) and Matrigel according to the manufacturer's instructions. Then, 1×10^5^ cells were seeded into the upper chamber with serum-free optiMEM medium. optiMEM with 10% FBS was added to the lower compartment. The cells were allowed to invade for 48 h. Matrigel membranes were fixed with ice-cold methanol and stained with a 0.1% crystal violet solution. The number of cells that migrated to the lower side was counted in five randomly selected fields under a light microscope.

### Statistical analysis

Quantitative data were analyzed using SPSS version 20.0 (IBM, USA) and expressed as the mean±s.d. Significant differences between groups were compared using ANOVAs and two-tailed t-tests. The level of statistical significance was set at *P*<0.05. All experiments, ANOVAs and two-tailed t-tests were repeated three times.

## SUPPLEMENTARY FIGURES AND TABLE


