# Supplementary material for: Negative feedback between TAp63 and Mir-133b mediates colorectal cancer suppression
Source: Oncotarget. 2016 Nov 23;7(52):87147–60. doi: 10.18632/oncotarget.13515 (PMC5349978; doi:10.18632/oncotarget.13515)
Supplement: Supplementary file 1 [file oncotarget-07-87147-s001.pdf]

# Negative feedback between TAp63 and Mir-133b mediates colorectal cancer suppression

## SUPPLEMENTARY FIGURES AND TABLE

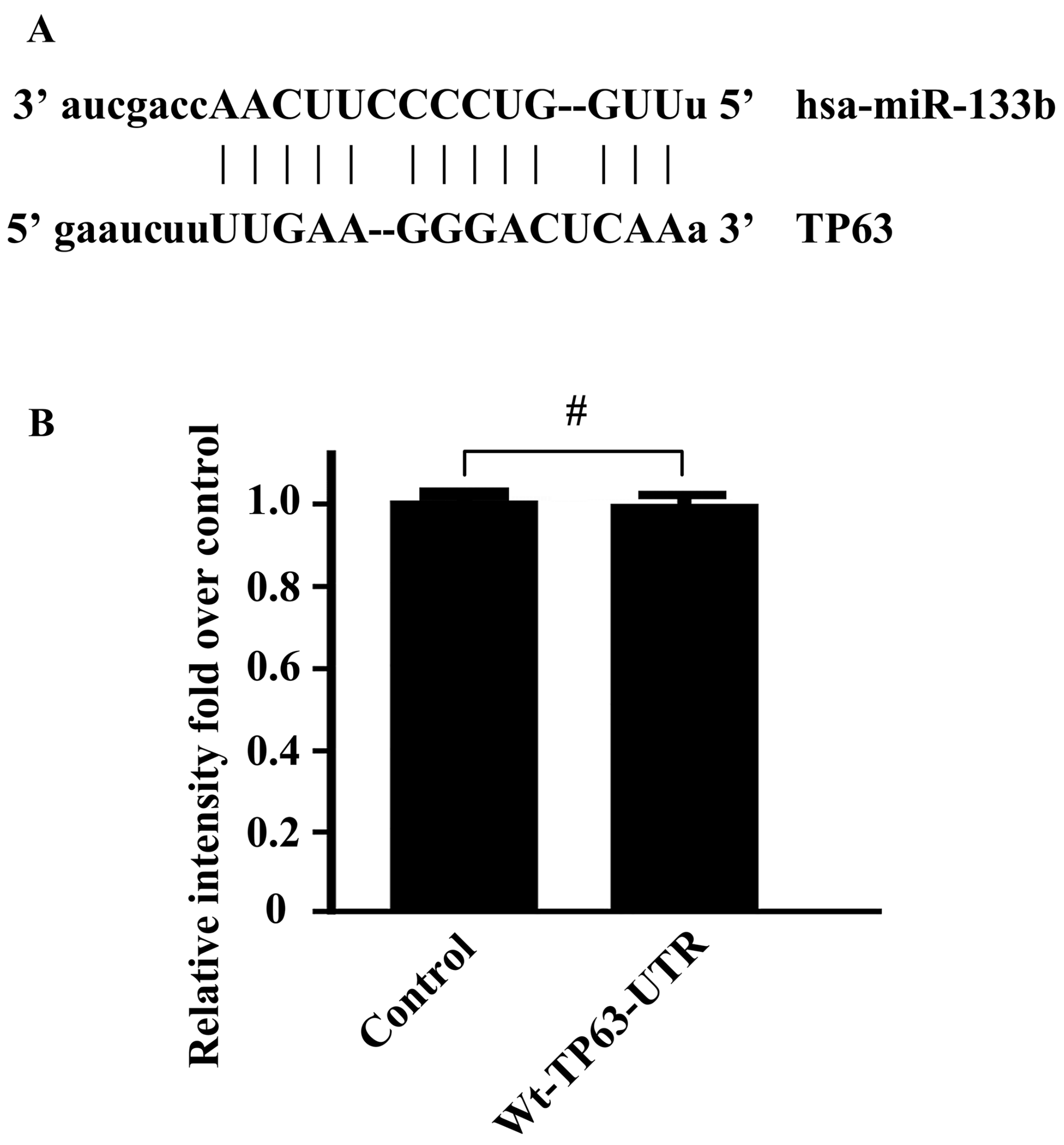


**Supplementary Figure S1: Luciferase assay. A.** position of the miR-133b target site along 3′-UTR of p63 **B.** HCT-116 cells were cotransfected with miR-133b vectors together with a firefly luciferase vector containing p63 3′-UTR and a Renilla luciferase control; the histogram indicates the relative luciferase unit (RLU) in different transfected cells; data represent the means ± SD of three different experiments analyzed in triplicate. [#]*P*>0.05.

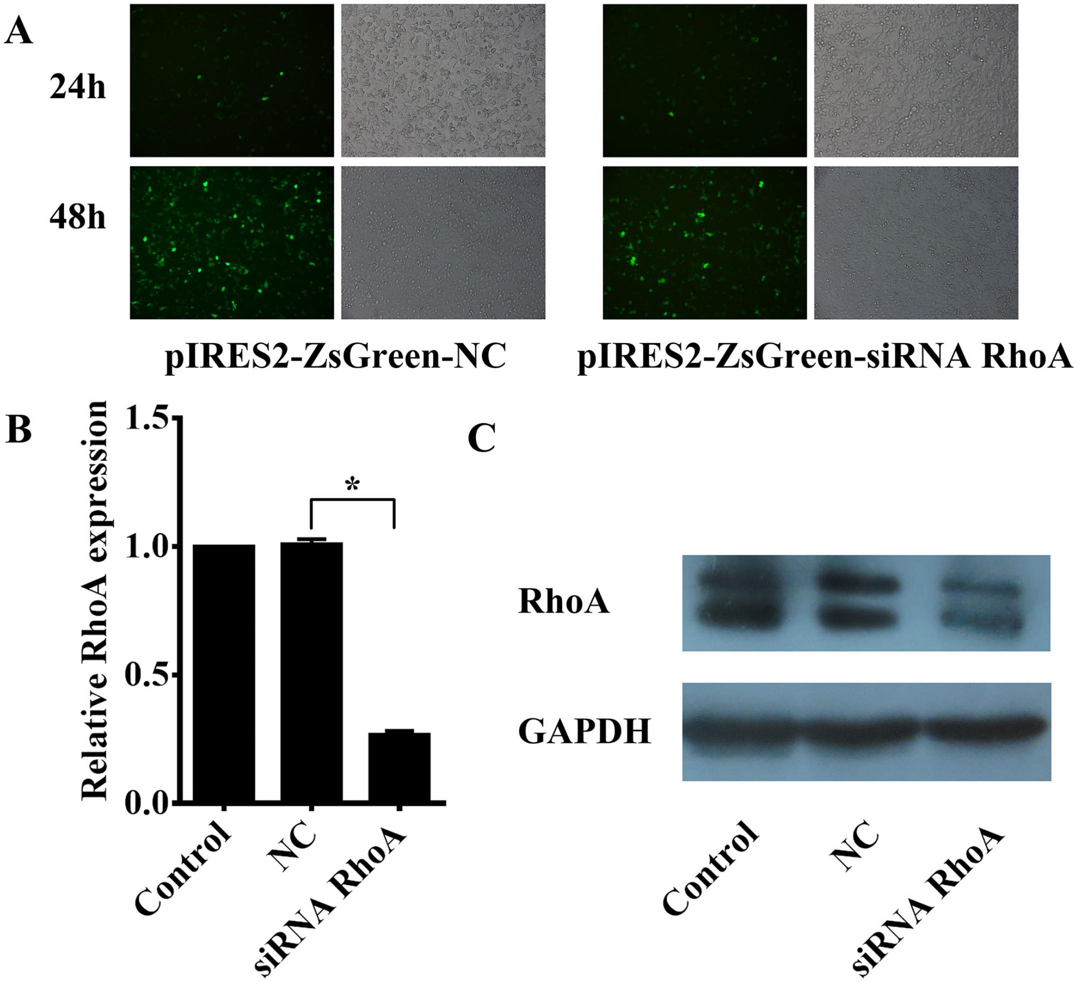


**Supplementary Figure S2: Silencing of RhoA by siRNA. A.** transfected with pIRES2-Zs-Green-NC (NC group) or pIRES2-Zs-Green-siRNA RhoA (siRNA RhoA group) for 24h and 48h exhibited green fluorescent signals under a fluorescence microscope and an inverted microscope, respectively. **B.** Expression of RhoA was measured by quantitative RT-PCR in Control group, NC group and siRNA RhoA group 48h after transfection. *$P < 0.05$. **C.** Expression of RhoA was measured by Western blot in Control group, NC group and siRNA RhoA group 48h after transfection.

**Supplementary Table S1: Subcutaneous tumor characteristics**

| Group | Tumor length | Tumor width | Tumor volume | Tumor weight | P Value |
|---|---|---|---|---|---|
| HCT-116 | 12.97±2.68 | 9.87±2.11 | 711.25±425.67 | 0.21±0.02 | |
| TAp63 | 10.00±1.45 | 8.36±1.19 | 379.85±163.74 | 0.18±0.01 | <0.05 |
| miR-133b | 10.08±1.07 | 7.23±1.93 | 260.32±184.96 | 0.14±0.01 | <0.05 |
| TAp63+sponge | 12.80±1.48 | 10.80±1.10 | 791.34±225.35 | 0.27±0.02 | <0.05 |

Data are presented as mean (SD), except where indicated. The level of statistical significance was set at $P<0.05$.
